# Genetic correlations between energy status indicator traits and female fertility in primiparous Nordic Red Dairy cattle

**DOI:** 10.1017/S1751731120000439

**Published:** 2020-03-13

**Authors:** T. Mehtiö, P. Mäntysaari, E. Negussie, A.-M. Leino, J. Pösö, E. A. Mäntysaari, M. H. Lidauer

**Affiliations:** 1 Production Systems, Natural Resources Institute Finland (Luke), Tietotie 2, FI-31600 Jokioinen, Finland; 2 Faba Co-op, PO Box 40, FI-01301 Vantaa, Finland

**Keywords:** mid-IR reflectance spectroscopy, heritability, fatty acid, beta-hydroxybutyrate, acetone

## Abstract

Inclusion of feed efficiency traits into the dairy cattle breeding programmes will require considering early lactation energy status to avoid deterioration in health and fertility of dairy cows. In this regard, energy status indicator (**ESI**) traits, for example, blood metabolites or milk fatty acids (**FAs**), are of interest. These indicators can be predicted from routine milk samples by mid-IR reflectance spectroscopy (**MIR**). In this study, we estimated genetic variation in ESI traits and their genetic correlation with female fertility in early lactation. The data consisted of 37 424 primiparous Nordic Red Dairy cows with milk test-day records between 8 and 91 days in milk (**DIM**). Routine test-day milk samples were analysed by MIR using previously developed calibration equations for blood plasma non-esterified FA (**NEFA**), milk FAs, milk beta-hydroxybutyrate (**BHB**) and milk acetone concentrations. Six ESI traits were considered and included: plasma NEFA concentration (mmol/l) either predicted by multiple linear regression including DIM, milk fat to protein ratio (**FPR**) and FAs C10:0, C14:0, C18:1 *cis*-9, C14:0 * C18:1 *cis*-9 (NEFA_FA_) or directly from milk MIR spectra (NEFA_MIR_), C18:1 *cis*-9 (g/100 ml milk), FPR, BHB (mmol/l milk) and acetone (mmol/l milk). The interval from calving to first insemination (**ICF**) was considered as the fertility trait. Data were analysed using linear mixed models. Heritability estimates varied during the first three lactation months from 0.13 to 0.19, 0.10 to 0.17, 0.09 to 0.14, 0.07 to 0.10, 0.13 to 0.17 and 0.13 to 0.18 for NEFA_MIR_, NEFA_FA_, C18:1 *cis*-9, FPR, milk BHB and acetone, respectively. Genetic correlations between all ESI traits and ICF were from 0.18 to 0.40 in the first lactation period (8 to 35 DIM), in general somewhat lower (0.03 to 0.43) in the second period (36 to 63 DIM) and decreased clearly (−0.02 to 0.19) in the third period (64 to 91 DIM). Our results indicate that genetic variation in energy status of cows in early lactation can be determined using MIR-predicted indicators. In addition, the markedly lower genetic correlation between ESI traits and fertility in the third lactation month indicated that energy status should be determined from the first test-day milk samples during the first 2 months of lactation.

## Implications

Including feed efficiency traits in dairy cow breeding programmes will require the energy status of cows in early lactation to be considered in order to avoid unfavourable effects on health and fertility. Novel indicators based on mid-infrared analysis of milk have been developed to determine the energy status of dairy cows. Evaluating genetic variation in energy status indicator traits and assessing their genetic correlations with fertility will serve as a basis for the development of new breeding and management strategies to enhance the efficiency, health and fertility of dairy cows.

## Introduction

In dairy cattle breeding programmes worldwide, there is a growing emphasis placed on functional traits to breed for health, efficiency, robustness and longevity (Egger-Danner *et al.*, 2015; Bastin *et al.*, 2016; Pryce *et al.*, 2016; König and May, 2019). In Nordic countries, the importance of reproductive and health traits was recognised already in the 1960s, and female fertility has been included into the breeding programmes for several decades (Philipsson and Lindhé, 2003). Currently, the inclusion of feed efficiency traits as breeding objectives has started. The relationship between energy balance and feed efficiency is reported to be strong and unfavourable especially in early lactation, indicating that selection for feed efficiency may lead to greater negative energy balance (Spurlock *et al.*, 2012; Liinamo *et al.*, 2015; Hurley *et al.*, 2018). Since severe negative energy status has been shown to have an unfavourable response on health and fertility (Leroy *et al.*, 2008; Bastin *et al.*, 2016; Pryce *et al.*, 2016), a breeding strategy for feed efficiency has to be carefully designed. Otherwise, there is a risk that cows in severe negative energy status might be selected as feed efficient animals. Thus, energy status should be considered in breeding programmes, and for this low-cost indicators that help determining the energy status of cows are needed.

High-yielding dairy cows typically tend to be in negative energy status during the early *postpartum* period due to a rapid increase in milk production and high energy demand which cannot be fulfilled by energy intake. To meet this energy demand, cows mobilise fatty acids (**FAs**) and glycerol from their adipose tissue. This mechanism is steered by complex hormonal regulation (Veerkamp *et al.*, 2003; Leroy *et al.*, 2008; Esposito *et al.*, 2014). Studies have indicated that there is genetic variation between cows in the efficiency of using metabolisable energy for milk production (Mehtiö *et al.*, 2018a), and high genetic merit cows tend to partition more energy to milk (Agnew and Yan, 2000; Veerkamp *et al.*, 2003). Veerkamp *et al.* (2003) in their review concluded that selection predominantly for high yield in dairy cows had affected the energy partitioning most likely due to genetic effects on the somatotropic axis, including growth hormone and IGF-1. The imbalance of hormones and metabolites and dysfunction of metabolic processes in severe negative energy status may predispose cows to metabolic diseases like ketosis and fatty liver syndrome and lead to decrease in fertility (Leroy *et al.*, 2008; Bastin *et al.*, 2016; Pryce *et al.*, 2016).

Nevertheless, accounting for energy status in genetic selection is difficult. Estimation of energy balance based on milk production and composition, DM intake, energy density of the diet and BW is possible, but its accuracy may be low due to accumulating measurement errors. Besides, DM intake is very rarely recorded on-farms. Using energy balance is disadvantageous in that highly efficient cows might apparently be in negative energy balance but are not necessarily in negative energy status and thus on a metabolically imbalanced state. McParland *et al.* (2012) predicted energy balance and body energy content using milk mid-IR reflectance spectroscopy (**MIR**) data and milk yield as predictor variables. However, they concluded that very high accuracy cannot be expected due to the difficulties in estimating energy balance and body energy content. Therefore, at the moment, evaluating energy status-related blood and milk metabolites as well as milk composition and milk FAs is of interest.

In the event of negative energy status, the carbohydrate insufficiency induces the use of adipose tissues. Adipose tissue metabolism is highly reactive and finely regulated, and there are numerous interactions between immune, endocrine and metabolic systems in dairy cows during early lactation (Chilliard *et al.*, 2000; Esposito *et al.*, 2014). However principally, mobilising adipose tissue increases the concentration of non-esterified FAs (**NEFAs**) in blood plasma. As the supply of NEFA is overloaded, the production of ketone bodies (acetoacetic acid, acetone and β-hydroxybutyrate (**BHB**)) in the liver increases (Chilliard *et al.*, 2000; Veerkamp *et al.*, 2003; Esposito *et al.*, 2014). Therefore, the aforementioned blood metabolites may serve as reliable indicators for the cow’s energy status. Energy status indicator (**ESI**) traits can be predicted using milk MIR spectra using prediction equations developed previously, for example, for blood NEFA (Mehtiö *et al.*, 2018b; Grelet *et al.*, 2019), blood and milk BHB (de Roos *et al.*, 2007; Belay *et al.*, 2017; Grelet *et al.*, 2019) and blood and milk acetone (de Roos *et al.*, 2007) concentrations. In addition, lipolysis results in changes in milk component ratios like fat to protein ratio (**FPR**). Therefore, FPR is one of the suggested ESI traits (Buttchereit *et al.*, 2010; Negussie *et al.*, 2013; Koeck *et al.*, 2014; Pryce *et al.*, 2016). Moreover, as the mobilisation of adipose tissue releases long-chain FAs and inhibits de novo FA synthesis in the mammary gland, it causes changes in milk FA profile. Thus, the proportion of FAs originating from adipose tissue (especially C16:0, C18:0 and C18:1 *cis*-9) in milk increases, and FA profile could also be used as another accurate ESI (Stoop *et al.*, 2009; Bastin *et al.*, 2011).

Earlier studies have shown that heritability estimates for milk FAs and metabolites in blood and milk vary during lactation (Oikonomou *et al.*, 2008a; Bastin *et al.*, 2011; Koeck *et al.*, 2014). Results from Oikonomou *et al.* (2008a) study indicated that the predictive capacity of blood NEFA and BHB concentrations ends 11 to 16 weeks after calving. Therefore, genetic evaluation for early lactation profile of body energy and blood metabolic traits could be possible with a single measurement obtained at any time during the first 2 to 3 months in lactation. Previous studies have also reported genetic relationships between energy status-related blood metabolites, milk FAs and fertility traits (Bastin *et al.*, 2016). For example, Bastin *et al.* (2012) found a moderate genetic correlation (0.39) between fertility trait days open and milk FA C18:1 *cis*-9 at 5 days in milk (**DIM**). Results from Koeck *et al.* (2014) indicated that selection for lower milk BHB in early lactation would lead to an improvement of several health and fertility traits. Also results from Oikonomou *et al.* (2008b) study indicated that blood NEFA and BHB concentration had an unfavourable genetic association with fertility traits. Leroy *et al.* (2005) even showed that high NEFA levels, associated with negative energy balance, are reflected in the follicular fluid of dominant follicles in dairy cows early *postpartum*. Results from their *in vitro* study revealed that the saturated long-chain FAs provoked an inhibition of maturation rate, leading to lower fertilisation, cleavage and blastocyst formation rates (Leroy *et al.*, 2005). The main objectives of this study were to estimate genetic variations in newly developed NEFA predictions, to compare these predictions to other ESI traits and to explore their genetic correlations with fertility in early lactation to assess the consequences of selection for ESI traits.

## Material and methods

### Traits

Blood plasma NEFA concentration (mmol/l) predicted directly from milk MIR spectra measured with a MilkoScan FT6000 spectrometer (Foss, Hillerød, Denmark) in Valio Ltd milk laboratory (Seinäjoki, Finland) was NEFA_MIR_. The prediction equation used for NEFA_MIR_ was developed in Mehtiö *et al.* (2018b). The data set for developing prediction equations consisted of 778 MIR spectral records of evening milk samples from 141 Nordic Red Dairy cows (**RDCs**) with blood NEFA samples collected on the same day. The coefficient of determination of cross-validation (**R**^**2**^**cv**) was 0.67 and the RMSE 0.17 mmol/l.

Blood plasma NEFA concentration predicted by multiple regression equation which included DIM, milk FPR and milk FAs C10:0, C14:0, C18:1 *cis*-9, C14:0 * C18:1 *cis*-9 (R^2^cv = 0.62 and RMSE = 0.18 mmol/l) was NEFA_FA_ (Mäntysaari *et al.*, 2019). Milk FA concentrations were predicted using equations by Soyeurt *et al.* (2011). For example, milk C18:1 *cis*-9 concentration was predicted with R^2^cv >0.97 and standard error of cross-validation 0.05 g/100 ml milk. Predictions for milk BHB and acetone concentrations were available from routine milk sample analyses (MilkoScan FT6000, FOSS, Hillerød, Denmark), and the calibration equations were based on de Roos *et al.* (2007).

Thus, the ESI traits considered in this study were NEFA_MIR_ and NEFA_FA_, milk FA C18:1 *cis*-9 (g/100 ml in milk), milk FPR, BHB (mmol/l milk) and acetone (mmol/l milk). The fertility trait considered in this study was interval from calving to first insemination (**ICF**). This trait is an important part of cows’ fertility complex. It is measured in days from calving and is indicative of a cow’s ability to resume cyclicity after calving and to manifest estrus behaviour (Muuttoranta *et al.*, 2019). In addition, ICF was chosen because of its susceptibility to negative energy status in early lactation. It is measured around the same time when cows are expected to be in negative energy status, and severe negative energy status is expected to cause a longer time for the first insemination after calving.

### Data

Four different sources of data were used to build the final data set used for the variance component estimation: milk MIR spectral data, milk recording test-day data, milk BHB and acetone data, and fertility data. Since May 2015, MIR spectra are automatically stored for the routine test-day milk samples analysed at Valio Ltd laboratory. By June 2018, there were over 2.7 million spectral readings collected, and NEFA_MIR_ and milk FAs, including C10:0, C14:0, C18:1 *cis*-9, were predicted for all these milk samples.

The NEFA_MIR_ and FA observations predicted from spectral readings were merged with the cow’s test-day information, which made it possible to predict NEFA_FA_ and also to calculate FPR. The ICF observations from national fertility evaluations were merged to the data with test-day records, NEFA and FA predictions. Milk BHB and acetone concentrations were collected for milk samples analysed at Valio Ltd laboratory during November 2015 to October 2017, and these records were also merged with the data. In the BHB and acetone data set, there were in total 105 164 records from primiparous RDC cows in early lactation (8 to 91 DIM).

Data edition for all studied variables included filtering for outliers and discarding observations that were greater than four SDs from the mean. For genetic analyses, milk BHB and acetone concentrations were log_e_-transformed to normalise their distribution. Before log transformation, a constant of 1.00 was added to BHB and acetone values to prevent negative and zero values during log-transformation.

In the Finnish routine test-day milk recording, milk fat and protein are sampled every second month, and thus it was decided to divide the first trimester into three periods: from 8 to 35 DIM, from 36 to 63 DIM and from 64 to 91 DIM. These three periods were considered as different but correlated traits. In this procedure, each cow had at least one of the period traits recorded. In case a cow had more than one observation in any period, the first one was kept. Some of the cows had records from more than one period, and the percentages of cows with one, two or three records in the data were 59.2%, 31.2% and 9.6%, respectively. To maintain a reasonable contemporary group sizes, herds with less than 24 NEFA_MIR_ records (i.e. approximately 8 records/month) were discarded from the data set. In addition, the largest herds with more than 150 NEFA_MIR_ records (1% of the data) were deleted to normalise the distribution of herd sizes. The final data set consisted of 37 424 primiparous RDC cows from 962 herds with 1, 2 or 3 ESI trait records in early lactation (i.e. before 92 DIM). A summary statistics for the final data set used in the analyses is presented in Table 1. For the genetic analyses, the pedigree was traced back to four generations from the cows with records and contained 121 542 informative animals.

**Table 1:** Summary statistics, variance components (genetic variance 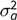 and residual variance 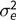) and heritability estimates (h^2^) of the data in (1) 8 to 35 days in milk (DIM), (2) 36 to 63 DIM and (3) 64 to 91 DIM for plasma non-esterified fatty acid (NEFA) concentration predicted from milk mid-IR spectra (NEFA_MIR_, mmol/l), plasma NEFA concentration predicted from milk fatty acids (NEFA_FA_, mmol/l), milk fatty acid C18:1 *cis*-9 (g/100 ml milk), milk fat to protein ratio (FPR), log-transformed beta-hydroxybutyrate (BHB, mmol/l milk), log-transformed acetone (mmol/l milk) from multivariate analyses of variables in three time windows and interval from calving to first insemination (ICF) from univariate analysis in primiparous Nordic Red Dairy cows

	Records no.	Mean	SD	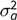	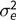	*h*^2^
NEFA_MIR1_	19 220	0.410	0.206	0.0058	0.0249	0.19
NEFA_MIR2_	19 329	0.286	0.159	0.0026	0.0163	0.14
NEFA_MIR3_	19 856	0.213	0.130	0.0021	0.0107	0.16
NEFA_FA1_	19 214	0.470	0.183	0.0039	0.0190	0.17
NEFA_FA2_	19 327	0.316	0.141	0.0014	0.0130	0.10
NEFA_FA3_	19 857	0.237	0.109	0.0010	0.0079	0.11
C18:1 *cis*-9_1_	19 193	1.083	0.355	0.0122	0.0741	0.14
C18:1 *cis*-9_2_	19 313	0.947	0.266	0.0047	0.0500	0.09
C18:1 *cis*-9_3_	19 850	0.867	0.214	0.0036	0.0328	0.10
FPR_1_	19 222	1.342	0.254	0.0043	0.0498	0.08
FPR_2_	19 261	1.316	0.239	0.0036	0.0445	0.07
FPR_3_	19 801	1.273	0.218	0.0035	0.0364	0.09
BHB_1_	12 396	0.055	0.061	0.0005	0.0026	0.16
BHB_2_	12 444	0.054	0.056	0.0004	0.0022	0.15
BHB_3_	12 851	0.050	0.050	0.0003	0.0017	0.15
Acetone_1_	12 310	0.037	0.093	0.0013	0.0060	0.18
Acetone_2_	12 438	0.036	0.079	0.0008	0.0045	0.15
Acetone_3_	12 848	0.028	0.069	0.0006	0.0034	0.15
ICF	32 479	83.35	28.86	17.38	636.51	0.03

### Genetic analyses

Multivariate linear mixed animal models were applied to ESI traits at 8 to 35, 36 to 63 and 64 to 91 DIM. In addition, univariate analyses were made for each lactation period separately. In matrix notation, the model can be written as:

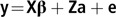

where **y** is a vector of observations; **β** is a vector of fixed effects of herd, year-month of the test-day for ESI traits and year-month of calving for ICF, age at calving and regression on DIM for ESI traits; **a** is a vector of random animal additive effects; **e** is a vector of random residuals; and **X** and **Z** are the corresponding design matrices. There were in total 962 herds, 38 year-month classes for test-days (from May 2015 to June 2018), 40 year-month classes for calvings and 9 age at calving classes, in which <22 and >30 were the first and last classes, respectively, and the other classes were single months. Random effects were assumed to be normally distributed with means equal to zero and the covariance matrix for **a**, var(**a**) = **G**_**0**_⊗**A**, where **G**_**0**_ is the covariance matrix for the random additive genetic effects and **A** is the additive genetic relationship matrix, and the covariance matrix for **e**, var(**e**) = **R**_**0**_⊗**I**, where **R**_**0**_ is the covariance matrix for the random residuals and **I** is an identity matrix.

Genetic analyses were made first within each ESI trait separately, applying a multi-trait model for all three periods to assess the genetic correlations between the periods as well as applying single-trait analyses within each period. Secondly, the correlations between the six different ESI traits and ICF were assessed applying multi-trait models within each period. Variance components were estimated using restricted maximum likelihood (REML) applying Average Information (AI-REML) method in DMU package (Madsen and Jensen, 2013). Standard errors for heritability estimates and genetic correlations were approximated using Taylor series expansions.

## Results

### Phenotypic description

**Figure 1:**
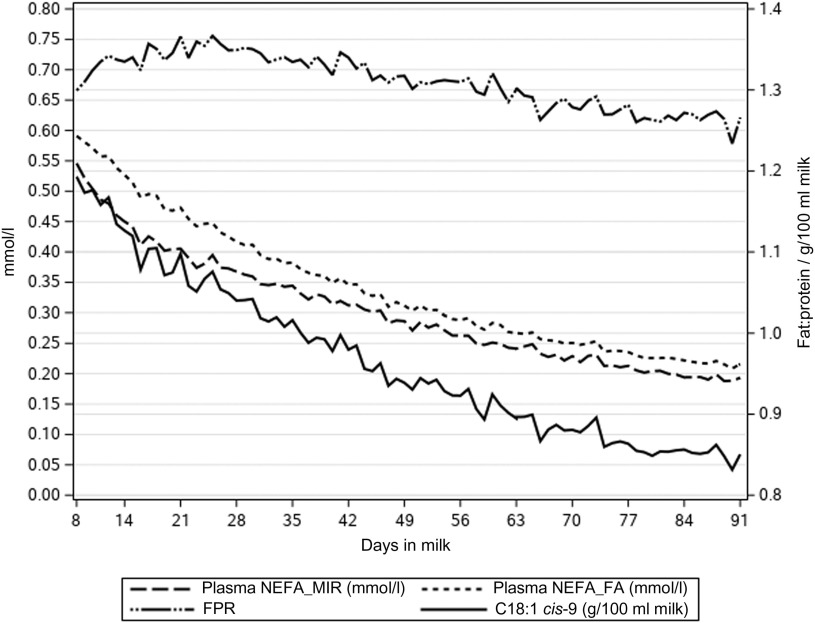
Lactation day means of plasma non-esterified fatty acid (NEFA) concentration predicted from milk mid-IR spectra (NEFA_MIR_, mmol/l), plasma NEFA concentration predicted from milk fatty acids (NEFA_FA_, mmol/l), milk fatty acid C18:1 *cis*-9 (g/100 ml milk) and milk fat to protein ratio (FPR) by days in milk in primiparous Nordic Red Dairy cows.

**Figure 2:**
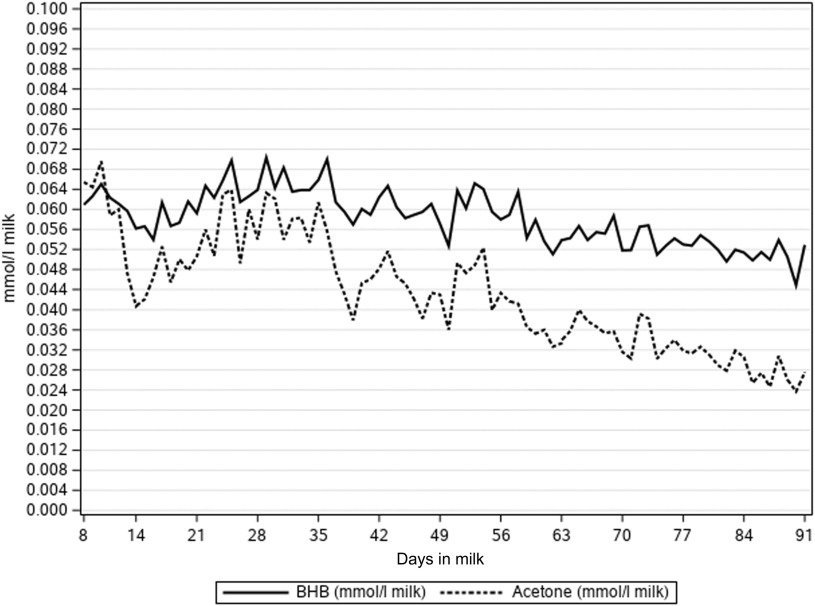
Lactation day mean milk beta-hydroxybutyrate (BHB, mmol/l) and acetone concentration (mmol/l) by days in milk in primiparous Nordic Red Dairy cows.

The NEFA concentrations predicted directly from milk MIR spectra (NEFA_MIR_) were lower than NEFA concentration predicted by multiple regression on DIM, milk FPR and FAs (NEFA_FA_) (Table 1, Figure 1). Figure 1 presents how the lactation day mean concentrations of NEFA_MIR_, NEFA_FA_, C18:1 *cis*-9 and FPR decreased as lactation progressed. Milk FA C18:1 *cis*-9 decreased from 1.19 to 0.85 g/100 ml milk during 8 to 91 DIM. Milk FPR stayed somewhat constant during the early lactation and varied from 1.30 to 1.26; however, a slight increase from 8 to 25 DIM and a decrease afterwards was observed. In Figure 2, mean milk BHB and acetone concentrations varied from 0.071 to 0.044 and from 0.070 to 0.024 mmol/l, respectively, during 8 to 91 DIM. These concentrations followed the same pattern, and milk acetone concentration stayed at a slightly lower level. Overall, the mean and SD at ESI traits were highest for the first period (from 8 to 35 DIM) and decreased as lactation progressed (Table 1). The ICF records were available for 32 479 cows in the data with NEFA prediction. The mean ICF was 83.35 days with SD of 28.86 days (Table 1).

### Genetic parameters of energy status indicator traits

Table 1 presents variance components and heritability estimates from multivariate analyses for all ESI traits in first 3 months of lactation. Heritability estimates varied during the time periods from 0.14 to 0.19, 0.10 to 0.17, 0.09 to 0.14, 0.07 to 0.09, 0.15 to 0.16 and 0.15 to 0.18 for NEFA_MIR_, NEFA_FA_, C18:1 *cis*-9, FPR, milk BHB and acetone, respectively. For all ESI traits, both the genetic and residual variances decreased during lactation. This resulted in slightly higher heritability estimates for all traits in the first period except for FPR. The heritability estimate for ICF was 0.03.

Genetic correlations between the three time periods for ESI traits are in Table 2. For all traits, genetic correlations between the first and the second period ranged from 0.86 to 0.89. In general, genetic correlations between the second and the third period were higher than between the first and second month, and ranged from 0.89 to 0.99 for all ESI traits. Genetic correlations were lowest between the first and the third period and ranged from 0.55 (C18:1 *cis*-9) to 0.84 (milk BHB).

**Table 2:** Genetic correlations between time periods (1) 8 to 35 days in milk (DIM), (2) 36 to 63 DIM and (3) 64 to 91 DIM with standard errors in parentheses for energy status indicator traits, plasma non-esterified fatty acid (NEFA) concentration predicted from milk mid-IR spectra (NEFA_MIR_, mmol/l), plasma NEFA concentration predicted from milk fatty acids (NEFA_FA_, mmol/l), milk fatty acid C18:1 *cis*-9 (g/100 ml milk), milk fat to protein ratio (FPR), log-transformed beta-hydroxybutyrate (BHB, mmol/l milk) and log-transformed acetone (mmol/l milk) from multivariate analyses in primiparous Nordic Red Dairy cows

	Lactation months		
Trait	1 * 2	1 * 3	2 * 3
NEFA_MIR_	0.87 (0.03)	0.69 (0.05)	0.92 (0.03)
NEFA_FA_	0.86 (0.04)	0.61 (0.07)	0.89 (0.04)
C18:1 *cis*-9	0.86 (0.05)	0.55 (0.08)	0.89 (0.04)
FPR	0.86 (0.06)	0.76 (0.07)	0.97 (0.04)
BHB	0.89 (0.05)	0.84 (0.06)	0.99 (0.03)
Acetone	0.87 (0.05)	0.77 (0.06)	0.98 (0.03)

### Genetic and phenotypic correlations between energy status indicator traits and fertility

Genetic correlations between ESI traits and fertility as well as heritabilities were estimated within each of the three periods separately. Results from the first period are presented in Table 3. Here, the strongest genetic correlations were between NEFA_FA_ and C18:1 *cis*-9 (0.95), milk BHB and acetone (0.95) and NEFA_FA_ and NEFA_MIR_ (0.91), and the lowest genetic correlations were between FPR and milk acetone, BHB and NEFA_MIR_ (from 0.30 to 0.44). Genetic correlations between ESI traits and ICF were moderate with 0.39 (±0.11) for NEFA_MIR_, 0.40 (±0.11) for NEFA_FA_, 0.36 (±0.12) for C18:1 *cis*-9, 0.18 (±0.14) for FPR, 0.38 (±0.12) for milk BHB and 0.33 (±0.12) for milk acetone. The lowest phenotypic correlations were between FPR and BHB, acetone and NEFA_MIR_ (from 0.31 to 0.50). The highest phenotypic correlations were between C18:1 *cis*-9 and NEFA_FA_ (0.96) and NEFA_FA_ and NEFA_MIR_ (0.88). In general, the phenotypic correlations between ICF and ESI traits ranged from 0.02 to 0.04.

**Table 3:** Heritability estimates^1^ (on the diagonal) and genetic correlations (above the diagonal) with standard errors in parentheses, and phenotypic correlations (below the diagonal; SE not available) for plasma non-esterified fatty acid (NEFA) concentration predicted from milk mid-IR spectra (NEFA_MIR_, mmol/l), plasma NEFA concentration predicted from milk fatty acids (NEFA_FA_, mmol/l), milk fatty acid C18:1 *cis*-9 (g/100 ml milk), milk fat to protein ratio (FPR), log-transformed beta-hydroxybutyrate (BHB, mmol/l milk) and log-transformed acetone (mmol/l milk) and interval from calving to first insemination (ICF) based on data from 8 to 35 days in milk in primiparous Nordic Red Dairy cows

	NEFA_MIR_	NEFA_FA_	C18:1 *cis*-9	FPR	BHB	Acetone	ICF
NEFA_MIR_	0.17 (0.02)	0.91 (0.02)	0.84 (0.03)	0.44 (0.08)	0.73 (0.05)	0.70 (0.05)	0.39 (0.11)
NEFA_FA_	0.88	0.17 (0.02)	0.95 (0.01)	0.59 (0.06)	0.71 (0.05)	0.70 (0.05)	0.40 (0.11)
C18:1 *cis*-9	0.83	0.96	0.14 (0.02)	0.64 (0.06)	0.58 (0.07)	0.56 (0.07)	0.36 (0.12)
FPR	0.50	0.71	0.78	0.08 (0.01)	0.38 (0.10)	0.30 (0.10)	0.18 (0.14)
BHB	0.62	0.59	0.54	0.41	0.17 (0.03)	0.95 (0.01)	0.38 (0.12)
Acetone	0.66	0.61	0.53	0.31	0.89	0.18 (0.03)	0.33 (0.12)
ICF	0.04	0.04	0.04	0.03	0.02	0.02	0.03 (0.01)

1 Heritability estimates are from single-trait analyses.

Within the second period, all estimates of genetic correlations within ESI traits dropped except for the correlations between FPR and BHB (0.44) and FPR and acetone (0.30) (Table 4). The highest genetic correlations were again between milk BHB and acetone (0.92), NEFA_FA_ and NEFA_MIR_ (0.87) and NEFA_FA_ and C18:1 *cis*-9 (0.83). Estimates of low to moderate genetic correlations were found between FPR and NEFA_MIR_ (0.23 ± 0.11), NEFA_FA_ (0.32 ± 0.10) and C18:1 *cis*-9 (0.40 ± 0.10). Genetic correlations between ESI traits and ICF were 0.43 (±0.11) for NEFA_MIR_, 0.28 (±0.13) for NEFA_FA_, 0.17 (±0.13) for C18:1 *cis*-9, 0.03 (±0.14) for FPR, 0.29 (±0.12) for milk BHB and 0.16 (±0.13) milk acetone. Thus, when compared to the correlations in the first period, genetic correlations between ESI traits and fertility were lower in the second period for all traits except for NEFA_MIR_. Phenotypic correlations were also in general lower in the second than in the first period within ESI traits but stayed at the same level between ESI and ICF.

**Table 4:** Heritability estimates^1^ (on the diagonal) and genetic correlations (above the diagonal) with standard errors (SEs) in parentheses, and phenotypic correlations (below the diagonal; SE not available) for plasma non-esterified fatty acid (NEFA) concentration predicted from milk mid-IR spectra (NEFA_MIR_, mmol/l), plasma NEFA concentration predicted from milk fatty acids (NEFA_FA_, mmol/l), milk fatty acid C18:1 *cis*-9 (g/100 ml milk), milk fat to protein ratio (FPR), log-transformed beta-hydroxybutyrate (BHB, mmol/l milk) and log-transformed acetone (mmol/l milk) and interval from calving to first insemination (ICF) based on data from 36 to 63 days in milk in primiparous Nordic Red Dairy cows

Trait	NEFA_MIR_	NEFA_FA_	C18:1 *cis*-9	FPR	BHB	Acetone	ICF
NEFA_MIR_	0.13 (0.02)	0.87 (0.03)	0.66 (0.06)	0.23 (0.11)	0.57 (0.07)	0.55 (0.07)	0.43 (0.11)
NEFA_FA_	0.84	0.10 (0.01)	0.83 (0.03)	0.32 (0.10)	0.69 (0.07)	0.68 (0.07)	0.28 (0.13)
C18:1 *cis*-9	0.77	0.94	0.09 (0.01)	0.40 (0.10)	0.53 (0.08)	0.52 (0.09)	0.17 (0.13)
FPR	0.45	0.69	0.76	0.07 (0.01)	0.44 (0.10)	0.28 (0.11)	0.03 (0.14)
BHB	0.55	0.51	0.47	0.36	0.13 (0.02)	0.92 (0.02)	0.29 (0.12)
Acetone	0.57	0.5	0.43	0.24	0.85	0.13 (0.02)	0.16 (0.13)
ICF	0.03	0.04	0.03	0.02	0.03	0.03	0.03 (0.01)

1 Heritability estimates are from single-trait analyses.

Within the third period, genetic correlations among all ESI traits have decreased (Table 5). The lowest genetic correlations were between FPR and NEFA_MIR_ (−0.02 ± 0.10), FPR and acetone (0.16 ± 0.11) and FPR and NEFA_FA_ (0.19 ± 0.10)_._ The highest genetic correlations were still between milk BHB and acetone (0.85), NEFA_FA_ and NEFA_MIR_ (0.79) and NEFA_FA_ and C18:1 *cis*-9 (0.71). Genetic correlations between ESI traits and ICF were 0.19 (±0.12) for NEFA_MIR_, 0.12 (±0.13) for NEFA_FA_, −0.02 (±0.14) for C18:1 *cis*-9, 0.01 (±0.14) for FPR, 0.18 (±0.13) for milk BHB and 0.13 (±0.13) for milk acetone. Phenotypic correlations have also decreased among ESI traits compared to the first and second periods but stayed at somewhat similar levels between ESI traits and ICF.

**Table 5:** Heritability estimates^1^ (on the diagonal) and genetic correlations (above the diagonal) with standard errors (SEs) in parentheses, and phenotypic correlations (below the diagonal; SE not available) for plasma non-esterified fatty acid (NEFA) concentration predicted from milk mid-IR spectra (NEFA_MIR_, mmol/l), plasma NEFA concentration predicted from milk fatty acids (NEFA_FA_, mmol/l), milk fatty acid C18:1 *cis*-9 (g/100 ml milk), milk fat to protein ratio (FPR), log-transformed beta-hydroxybutyrate (BHB, mmol/l milk) and log-transformed acetone (mmol/l milk) and interval from calving to first insemination (ICF) based on data from 64 to 91 days in milk in primiparous Nordic Red Dairy cows

Trait	NEFA_MIR_	NEFA_FA_	C18:1 *cis*-9	FPR	BHB	Acetone	ICF
NEFA_MIR_	0.16 (0.02)	0.79 (0.04)	0.48 (0.07)	−0.02 (0.10)	0.41 (0.09)	0.39 (0.08)	0.19 (0.12)
NEFA_FA_	0.76	0.12 (0.02)	0.71 (0.05)	0.19 (0.10)	0.47 (0.09)	0.54 (0.08)	0.12 (0.13)
C18:1 *cis*-9	0.66	0.90	0.10 (0.01)	0.42 (0.09)	0.23 (0.11)	0.26 (0.10)	−0.02 (0.14)
FPR	0.32	0.62	0.74	0.10 (0.02)	0.34 (0.10)	0.16 (0.11)	0.01 (0.14)
BHB	0.44	0.40	0.34	0.34	0.14 (0.02)	0.85 (0.03)	0.18 (0.13)
Acetone	0.44	0.39	0.28	0.16	0.83	0.15 (0.02)	0.13 (0.13)
ICF	0.02	0.03	0.02	0.02	0.02	0.01	0.03 (0.01)

1 Heritability estimates are from single-trait analyses.

## Discussion

Several countries have now started working to include feed efficiency into dairy cattle breeding programmes, and it will even become more common as and when more feed efficiency data become available. Previous studies have shown that feed efficiency traits have unfavourable correlation with calculated energy balance (Spurlock *et al.*, 2012; Liinamo *et al.*, 2015; Hurley *et al.*, 2018), and failure to account for mobilisation of body reserves, for example, by using BW change, may result in selection for negative energy status. Thus, consideration of energy status as breeding goal is needed. This is to ensure that cows in severe negative energy status in early lactation will not be favoured and its unfavourable effects on health and fertility avoided. Nowadays, MIR spectrometry can be used to obtain novel milk phenotypes like ESI traits (De Marchi *et al.*, 2014; Bastin *et al.*, 2016; Pryce *et al.*, 2016; König and May, 2019). However, estimates of the genetic correlations between novel ESI traits and fertility, as well as with production and feed efficiency traits, are still lacking. Here, we used ICF as a fertility trait. The hypothesis was that ICF is prolonged by negative energy status, and thus this fertility trait was used as a reference trait to validate ESI traits. This makes ESI traits comparable among each other by assessing their genetic correlations with ICF. As all other fertility traits, also ICF is influenced by management. However, voluntarily prolonging time from calving to first service in high-yielding cows is not as common in Finland as in some other countries.

In this study, blood plasma NEFA concentration was predicted either directly from milk MIR spectra (NEFA_MIR_) or by multiple regression based on DIM, milk FPR and milk FAs (NEFA_FA_). Predicted NEFA_FA_ was on higher level during the early lactation compared to the predicted NEFA_MIR_. However, the genetic variance and heritability estimates were higher for NEFA_MIR_. For both the traits, the cross-validation accuracies during the development of prediction equations were at a reasonable level, but could still be improved as the prediction equations are updated with larger and more comprehensive data sets. In this study, only primiparous cows were considered, because developed prediction equations for NEFA were mainly based on observations from first parity. Plasma NEFA concentration is more rarely explored in genetic studies. This is because laborious blood sampling inhibits the collection of very large data sets, and hence studies on MIR-predicted NEFA are still scarce. However, Oikonomou *et al.* (2008a) analysed data from 365 cows with weekly measured blood metabolites from the first 3 months of lactation and monthly thereafter until the end of lactation by fitting random regression model. The estimated heritability for NEFA ranged from 0.08 to 0.35 and for BHB from 0.08 to 0.40, and the genetic variance for both traits was particularly high during first weeks of lactation (Oikonomou *et al.*, 2008a). These heritabilities are on the same level with the heritabilities estimated in our study. Here, we found heritability estimates for predicted NEFAs ranging from 0.10 to 0.19 and for BHB from 0.13 to 0.17. In our study, the genetic correlations between NEFA predictions and ICF varied from 0.12 to 0.43, depending on the NEFA trait and lactation month. Oikonomou *et al.* (2008b) reported genetic correlations between blood NEFA and several fertility traits ranging from −0.17 (between blood NEFA and first-lactation first-service conception rate) to 0.42 (between blood NEFA and presence of metritis).

Fat to protein ratio is a readily available trait, as milk fat and protein contents are easily extracted from the routine national recording schemes. In our study, heritability estimates for FPR were lower (from 0.07 to 0.10) than those presented in previous studies. Negussie *et al.* (2013) reported heritability estimates of 0.16, 0.19 and 0.23 at 30, 60 and 110 DIM, respectively, and Koeck *et al.* (2014) reported a heritability estimate of 0.12 for FPR on the first test-day. Negussie *et al.* (2013) reported genetic correlations between FPR and ICF of 0.28 when FPR was recorded at 30 DIM and 0.14 when FPR was recorded at 60 DIM, and these estimates are higher than the genetic correlations estimated in our study (from 0.03 to 0.18). Low heritability estimates found in this study for both traits, FPR and ICF, made estimating covariances between the traits difficult and resulted in high standard errors of genetic correlations.

Bastin *et al.* (2011) reported markedly varying genetic correlations between C18:1 *cis*-9 and other milk FAs during the first 100 DIM. This indicates that there is a relationship between the energy status of the cow and its milk composition, and that C18:1 *cis*-9 could be an indicator for mobilisation of body reserves (Bastin *et al.*, 2011). In our study, heritability estimates for milk FA C18:1 *cis*-9 ranged from 0.09 to 0.14, and genetic correlations with ICF varied from −0.02 to 0.36. These results are in line with Bastin *et al.* (2012) who reported a heritability estimate of 0.13 for C18:1 *cis*-9 at 5 DIM, and the estimate increased as lactation progressed. In their study, the genetic correlation was 0.39 (±0.12) between fertility trait days open and C18:1 *cis*-9 at 5 DIM, and the correlation decreased as lactation progressed and turned to negative at 95 DIM (Bastin *et al.*, 2012).

During negative energy status, the production of ketone bodies (acetoacetic acid, acetone and BHB) in liver is increased due to the elevation of NEFA concentration in blood (Esposito *et al.*, 2014). Van der Drift *et al.* (2012) assessed genetic parameters for plasma BHB and milk BHB, based on FOSS calibration equations. For the first 3 months of lactation, they reported moderate genetic correlation (0.52) between the traits. Heritability estimates for milk BHB ranged from 0.13 to 0.17 in our study, which are in line with estimates in the literature. For example, van der Drift *et al.* (2012) reported heritability estimates of 0.17 and 0.16 for plasma and milk BHB, respectively. Based on FOSS calibration equations, Koeck *et al.* (2014) estimated genetic parameters of milk BHB, and FPR from test-day milk samples recorded from 5 to 100 DIM in first lactation Canadian Holstein cows. They reported heritability estimates ranging from 0.14 to 0.29 for BHB across early lactation and the genetic correlation of 0.49 between milk BHB and FPR (Koeck *et al.*, 2014). Using random regression models, Lee *et al.* (2016) reported heritability estimates for milk BHB varying from 0.11 to 0.07 during early lactation between 4 and 90 DIM. In our study, the genetic correlation between BHB and ICF decreased from the first month (0.38) to the third month (0.18). Oikonomou *et al.* (2008b) found moderate genetic correlations between blood BHB and several fertility traits ranging from −0.65 (between blood BHB and conception rate in the first 305 days of first lactation) to 0.56 (between blood BHB and number of inseminations per conception).

In this study, the heritability estimates for acetone ranged from 0.13 to 0.18. This is somewhat close to the heritability estimate of 0.10 reported by van der Drift *et al.* (2012). Lee *et al.* (2016) estimated genetic parameters for milk acetone using random regression models and reported average heritability of 0.29 across lactation. Heritability estimates ranged from around 0.15 to 0.30 in early lactation (from 4 to 90 DIM) (Lee *et al.*, 2016). In our study, we found high correlations between milk BHB and milk acetone concentrations (from 0.95 to 0.83), which is in line with estimates of van der Drift *et al.* (2012). They reported a genetic correlation of 0.90 in early lactation (from 5 to 60 DIM).

Oikonomou *et al.* (2008a) suggested that the predictive capacity of NEFA ends from 11 to 16 weeks after calving. This is in line with the results of our study which confirmed a decreasing trend in the genetic correlations with the progress in lactation. Moreover, the genetic correlation between ESIs and fertility dropped rapidly as lactation progressed. Therefore, we suggest evaluating energy status using the first test-day result within 2 months *postpartum*.

All studied indicators in the present study were promising candidates for evaluating energy status of a cow. Newly developed NEFA_MIR_ and NEFA_FA_ were on the same level with C18:1 *cis*-9, BHB and acetone on heritability and genetic correlation with ICF, especially during the first month of lactation. The best suitable ESI trait should be selected according to the milk test-day recording design. If for some cows the first milk MIR spectral readings are only available after the first month in lactation, that is, milk samples are collected bi-monthly, then based on our results the use of NEFA_MIR_, NEFA_FA_ or BHB in milk would be recommended. However, during the second and third month of lactation, NEFA_MIR_ had slightly higher heritability and especially higher correlation with ICF during the third month of lactation. Also, the higher genetic variance is supporting the use of NEFA_MIR_. Therefore, based on this data set and predictions, we suggest the use of NEFA_MIR_ as an indicator, especially if the records are not available from the first lactation month. The relatively high genetic correlations (from 0.70 to 0.73) between NEFA predictions and BHB and acetone indicated that variation in these indicators may be largely explained by the same variation in the milk MIR spectra. However, in the second time period, the genetic correlations decreased to 0.57 between NEFA_MIR_ and BHB and 0.55 between NEFA_MIR_ and acetone, but were still 0.69 between NEFA_FA_ and BHB and 0.68 between NEFA_FA_ and acetone. In the third period, the genetic correlations were even lower between the traits. Thus, in the very early lactation, NEFA predictions, BHB and acetone are explaining a lot of the same variation. However, later in the lactation, there are more differences between two NEFA predictions and also in all other ESI traits. This is an area for further investigation. Nevertheless, the indications are all that during the period later than in the first month of lactation an ideal phenotype of energy status could be a combination of ESI traits as proposed by Grelet *et al.* (2019).

## Conclusions

In the future, the inclusion of feed efficiency traits into breeding programmes will also require consideration of energy status to prevent the decline in health and fertility of dairy cows. Energy status indicators, for example, blood metabolites or milk FAs, can be predicted using routine MIR analysis of milk samples. In this study, heritability estimates for ESI traits were from low to moderate during the first 3 months of lactation. Genetic correlations between ESI traits and ICF were moderate in the first 2 months period after calving and decreased afterwards, and thus energy status should be recorded from the first test-day result within 2 months *postpartum*. These results indicate that energy status in early lactation is possible to evaluate using MIR-based indicators.

## Software and data repository resources

Neither software nor data are deposited in an official repository.
